# Effects of Gender on the Association of Urinary Phthalate Metabolites with Thyroid Hormones in Children: A Prospective Cohort Study in Taiwan

**DOI:** 10.3390/ijerph14020123

**Published:** 2017-01-29

**Authors:** Te-I Weng, Mei-Huei Chen, Guang-Wen Lien, Pai-Shan Chen, Jasper Chia-Cheng Lin, Cheng-Chung Fang, Pau-Chung Chen

**Affiliations:** 1 Institute of Forensic Medicine, College of Medicine, National Taiwan University, Taipei 10051, Taiwan; wengtei2@ntu.edu.tw (T.-I.W.); paishanchen@ntu.edu.tw (P.-S.C.); 2 Forensic and Clinical Toxicology Center, National Taiwan University College of Medicine and National Taiwan University Hospital, Taipei 10051, Taiwan; conrad@ntu.edu.tw; 3 Department of Emergency Medicine, National Taiwan University Hospital, Taipei 10002, Taiwan; lovingbest@yahoo.com.tw; 4 Institute of Occupational Medicine and Industrial Hygiene, National Taiwan University College of Public Health, Taipei 10055, Taiwan; miffych@gmail.com (M.-H.C.); f92844007@gmail.com (G.-W.L.); 5 Department of Environmental and Occupational Medicine, National Taiwan University College of Medicine and National Taiwan University Hospital, Taipei 10051, Taiwan; 6 Department of Public Health, National Taiwan University College of Public Health, Taipei 10055, Taiwan

**Keywords:** phthalates, thyroid hormones, children, gender

## Abstract

Phthalates are considered endocrine disruptors. Our study assessed the gender-specific effects of phthalate exposure on thyroid function in children. In total, 189 Taiwanese children were enrolled in the study. One-spot urine and blood samples were collected for analyzing 12 phthalate metabolites in urine and thyroid hormones. The association between urinary phthalate metabolites and serum thyroid hormones was determined using a generalized linear model with a log link function; the children were categorized into groups for analysis according to the 33rd and 66th percentiles. The data were stratified according to gender and adjusted for a priori defined covariates. In girls, a positive association existed between urinary di-2-ethylhexyl phthalate (DEHP) metabolites (mono-(2-ethylhexyl) phthalate, mono-(2-ethyl-5-oxohexyl) phthalate, and mono-(2-ethyl-5-hydroxyhexyl) phthalate) and free thyroxine (T4). In boys, urinary dibutyl phthalate (DBP) metabolites (mono-i-butyl phthalate and mono-n-butyl phthalate) were positively associated with free triiodothyronine (T3). After categorizing each exposure into three groups, urinary DEHP metabolites were positively associated with free T3 levels in boys. Our results suggested that DEHP is associated with free T4 in girls and that DBP is associated with free T3 in boys. Higher DEHP metabolite concentrations exerted larger effects on free T3 in boys. These results reveal the gender-specific relationships between phthalate metabolites and thyroid hormones.

## 1. Introduction

Phthalates have been used as plasticizers for a long time. Humans are exposed to phthalates through multiple routes [1,2]. Although phthalate esters are rapidly metabolized into monoesters and are then further oxidized into oxidative metabolites in the human body, they have been associated with numerous serious health problems including endocrine disruption, reproductive and developmental toxicity, and respiratory allergy [3,4]. During the last decade, concerns have grown regarding the health risks associated with phthalate exposure, particularly in susceptible populations such as pregnant women and children [4].

Phthalate esters are a family of chemicals whose structure only varies in the length of their ortho-positioned hydrocarbon chain “arms”. Chain length varies from 1 to >10 carbons, in linear or branched format. Some phthalates—including dibutyl phthalate (DBP), di-2-ethylhexyl phthalate (DEHP), and diisobutyl phthalate—are classified in the EU as reproductive toxicants. Other phthalates are considered endocrine disruptors that cause anti-androgenic, estrogenic, and anti-thyroid activity in animal studies [5,6,7]. Thyroid hormones are considered crucial for maintaining normal physiological function during development. Many in vitro and in vivo studies have reported that phthalates have functions similar to thyroid hormones and the ability to bind to thyroid receptors and thus affect thyroid homeostasis [8,9,10].

Although studies have reported the effects of certain phthalates on the thyroid profile, the mechanisms underlying the chemical influence of particular phthalates on particular thyroid hormones remain inconclusive. Moreover, numerous toxicology studies have been conducted only in animals [11,12,13,14]. Serum levels of free thyroxine (T4) and total triiodothyronine (T3) in adult men were negatively associated with concentrations of DEHP metabolites [15]. In pregnant women, urinary concentrations of DBP metabolites were negatively correlated with serum levels of free and total T4 [16]. Phthalate exposure in children is probably higher than that in adults considering intake–body weight correlation [17]. Furthermore, adverse health outcomes caused by environmental chemicals may be of greater relevance in children because appropriate serum levels of thyroid hormones are critical for growth and neurological development. Most human studies have focused on pregnant women, infants, toddlers, adolescents, or adults. The understanding of phthalates action on thyroid hormones in prepubescents, however, is limited. 

A major scandal involving the illegal usage of phthalates as clouding agents in food and other products occurred in Taiwan in April–May 2011, with one study investigating the relationship between DEHP-tainted foodstuffs exposure and thyroid function in possibly affected children and adolescents [18]. After this scandal, the Taiwanese government introduced legislation banning the addition of phthalates to food. We conducted a study to assess the exposure to eight phthalates of prepubescents by measuring their metabolites in urine samples and hence determine the effect of prepubertal phthalate exposure on subsequent thyroid function.

## 2. Materials and Methods

### 2.1. Study Participants and Design

The study subjects were selected from two Taiwan Birth Panel Study (TBPS) longitudinal birth cohort studies. The details of both cohorts were described elsewhere [19]. Briefly, during 2004 and 2005, the TBPS recruited 486 births from one medical center, one regional hospital, and two clinics in the Taipei metropolitan area. The TBPS conducted follow-up studies when the children were 4 months, 6 months, 1 year, 2 years, 3 years, 5 years, 7 years, and 9–10 years old. Informed consent was obtained from all participants before enrollment, and the institutional review board of Taiwan National University Hospital approved the study (Project Identification Code: 201112137RIC). Parents were interviewed by trained interviewers using a structured questionnaire to record parental demographics and socioeconomic status, lifetime residential history, and environmental exposure history. A total of 189 biological samples were collected when the participants were 9–10 years old (from June 2013 to January 2014) after excluding data and loss during follow-ups. There were no significant differences between the basic demographics of our final study population and the initial study population (data not shown).

The collected blood samples were separated into plasma and blood cells. Urine and plasma samples were stored at −20 °C until further processing for laboratory analysis. 

### 2.2. Measurement of Phthalate Metabolite Concentrations

We developed a rapid and sensitive ultra-high-performance liquid chromatography (UPLC)–tandem mass spectrometry (MS/MS) method using the SCIEX Qtrap 6500 LC/MS/MS system (SCIEX, Framingham, MA, USA) to determine concentrations of 12 phthalate metabolites in urine. All laboratory analyses were performed by investigators blinded to the participants’ characteristics.

Concentrations of 12 phthalate metabolites were measured using UPLC–MS/MS: monomethyl phthalate (MMP), a dimethyl phthalate metabolite; monoethyl phthalate (MEP), a diethyl phthalate (DEP) metabolite; mono-3-carboxypropyl phthalate (MCPP) and mono-n-octyl phthalate (MOP), di-n-octyl phthalate metabolites; mono-n-butyl phthalate (MnBP) and monoisobutyl phthalate (MiBP), DBP metabolites; monocyclohexyl phthalate (MCHP), a dicyclohexyl phthalate metabolite; monobenzyl phthalate (MzBP), a benzylbutyl phthalate metabolite; mono-2-ethylhexyl phthalate (MEHP), mono-2-ethyl-5-oxohexyl phthalate (MEOHP), and mono-2-ethyl-5-hydroxyhexyl phthalate (MEHHP), DEHP metabolites; and monoisononyl phthalate (MiNP), a diisononyl phthalate metabolite. A 100-µL sample of urine was mixed with 20 μL of ammonium acetate buffer (1 M, pH = 6.5) and 10 µL of β-glucuronidase (200 U/mL, *Escherichia coli* K12). After enzymatic hydrolysis, the samples were mixed with 20 μL of 20 μg/L internal standard solutions (13C4-MMP, 13C4-MBP, and 13C4-MEHP; purity >99% in methyl tert-butyl ether, Cambridge Isotope Laboratories, Inc., Andover, MA, USA), acidified using 40 µL of sodium acetate (pH = 4.5) and 10 µL of deionized water, mixed until homogeneous, and analyzed using UPLC–MS/MS.

The detailed methods for analyzing urinary phthalate metabolites were described by Lien et al. (submitted, under revision). Recoveries and relative standard deviations were evaluated using three replicate fortifications of human urine samples with 0.01 μg/mL of standard solution. The recoveries of MMP, MEP, MiBP, MnBP, MBzP, MEHP, MEHHP, MEOHP, MCHP, MCPP, MOP, and MiNP were 102.1%, 112.1%, 85.2%, 79.0%, 87.4%, 88.2%, 108.3%, 105.2%, 84.6%, 87.7%, 111.7%, and 106.1% and their standard deviations were 7.6%, 12.5%, 9.6%, 9.4%, 6.7%, 5.9%, 8.2%, 7.8%, 6.6%, 7.3%, 5.3%, and 8.17%, respectively. The limit of quantitation (LOQ) for MMP, MEP, MiBP, MnBP, MBzP, MEHP, MEHHP, MEOHP, MCHP, MCPP, MOP, and MiNP were 0.1, 0.5, 0.5, 0.5, 0.05, 0.5, 0.05, 0.05, 0.5, 0.5, 0.5, and 0.5 μg/L, respectively.

Urine samples were collected from the participants in the morning when they were aged 9–10 years and were stored at −20 °C until analysis. For concentrations less than the detection limits, we assigned a value of half the LOQ. The samples with phthalate metabolite concentrations higher than the LOQ by more than 50% were included in this study. All samples were analyzed in triplicate.

Urine creatinine levels were analyzed using an enzymatic assay according to the manufacturer’s instructions (Cayman Chemical, Ann Arbor, MI, USA; Cayman Chemical Company, 2012). All phthalate metabolite concentrations were adjusted for urine creatinine levels and expressed as micrograms per gram of creatinine.

### 2.3. Thyroid Hormone Measurement

The endocrine profile included free T4, total T4, free T3, total T3, and thyroid-stimulating hormone (TSH), all measured using chemiluminescence immunoassay (Elecsys 2010 and Modular Analytics E170, Roche Diagnostics, GmbH, Indianapolis, IN, USA ). All serum thyroid hormone levels were detectable.

### 2.4. Statistical Analysis

Statistical analyses were performed using SAS 9.2 and R 3.1.0 (SAS Institute, Cary, NC, USA). 

The mean ± standard deviation or number (frequency) was tabulated to describe demographic characteristics, urinary metabolites, and serum thyroid profiles, as appropriate. Because urine was collected as spot samples, phthalate concentrations were adjusted by dividing them by the creatinine concentration in analyses. 

None of the compounds was linearly associated with the thyroid hormones. We used two methods to assess the relationship between various phthalate metabolites and thyroid function. First, we used the generalized linear model with a log link function to estimate the relationship thyroid hormones and other potential confounders. We considered the following potential confounders according to a review of relevant literature maternal age (>34 years), maternal education (higher than a college degree), family income [>1,500,000 New Taiwan dollars (NT$), and birth weight (>2500 g). Second, each exposure was categorized into one of three groups according to the 33rd and 66th percentiles to compare differences in thyroid hormone levels using nonparametric one-way analysis of variance with the Kruskal–Wallis test. To determine the gender-specific effects of exposure, models were stratified according to gender. All statistical analyses were performed with both crude, creatinine-corrected phthalate concentrations, and after adjustment for potential confounders. *p* < 0.05 indicated statistical significance.

## 3. Results

### 3.1. Participant Characteristics

The demographics of the 189 participants are presented in Table 1. All urine samples contained measurable amounts of metabolites of different phthalates. The distributions of crude and creatinine-corrected phthalate metabolites concentrations are listed in Table 2.

The samples with phthalate metabolite concentrations more than the LOQ by more than 50% were included in this study. We analyzed 12 phthalate metabolites but only SIX metabolites satisfied this criterion. MnBP, MEHP, MEP, MiBP, MEOHP, and MEHHP were detected in almost all urine samples, 86.3%–100% (Table 2). When we excluded the cases where urine phthalate metabolite concentration was lower than LOQ, the effects of the phthalate metabolites on the thyroid function in the 9–10-year-old children were similar to our study design. We defined a base value as half of the lower limit of quantitation (1/2LOQ). For sample data where the concentration is below LOQ, it is replaced the base value (data not shown). This method did not cause any bias in our statistical analysis.

**Figure 1:**
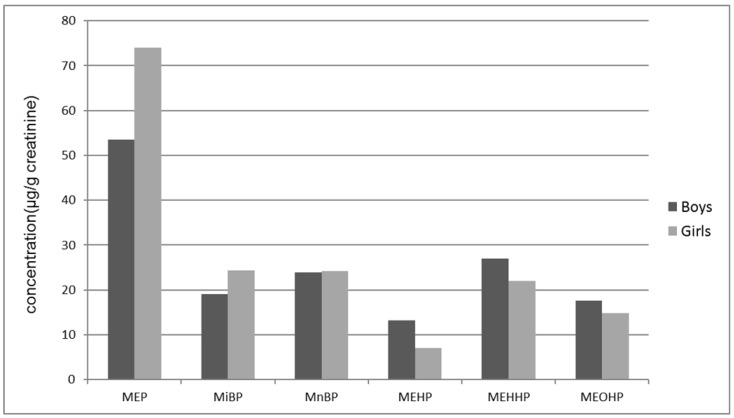
Urine phthalate metabolite concentrations (geometric mean).

Thyroid hormone levels are presented in Table 3. The thyroid hormones were not normally distributed. MEP, MiBP, MnBP, MEHP, MEHHP, MEOHP, and were detected in almost all urine samples (86.3%–100%; Table 2). The median concentration of MEP was the highest, followed by MEHHP and MnBP (Table 2, Figure 1). Samples from one girl revealed an extremely high MEP concentration (1407 ng/mL) that remained high after creatinine correction. No significant differences existed between boys and girls regarding urinary concentrations of various phthalate metabolites (Figure 1). 

### 3.2. Association between Concentrations of Urine Phthalate Metabolites and Thyroid Hormones

Coefficients (βs) and 95% confidence intervals (CIs) are presented in Table 4 as crude data and after correction for creatinine and adjustments for confounders. The model yielded similar results before and after covariates adjustment (Table 4, Table 5 and Table 6, Supplementary Tables S1–S3). We discuss the results of crude and creatinine-correction analysis after adjustment for confounders.

We also examined the correlations in these different phthalate metabolites (data not shown). Strong correlation was between MEHHP and MEOHP concentrations (Pearson’s *r* > 0.90). According to the DEHP metabolic pathway, the first step in metabolism of DEHP results in the formation of MEHP. MEHHP and MEOHP, two other DEHP metabolites, are formed through the oxidative metabolism of MEHP. Therefore, it is expected that MEHHP and MEOHP would be strongly correlated. We used MEHHP and MEOHP interaction variables in our analysis, but the interaction term was not significant for our thyroid function models (data not shown). Therefore, only the analytical results of the original model are presented.

According to model estimates, TSH levels increased by 16% per unit increase in urine MnBP in the crude analysis (95% CIs = 0.0001, 0.0031) after adjustment for confounders. However, the relationship between phthalates metabolites and thyroid hormones was not be significant in the creatinine-corrected analysis after confounder adjustment. 

We next performed a gender-stratified analysis (Table 4). Crude or creatinine analysis of the boygroup, after adjustment for confounders, revealed that free T3 levels increased with an increase in urine DBP metabolites (95% CIs [crude: MnBP = 0.00001, 0.0013, and ΣDBP = 0.00001, 0.0008]; [creatinine correction: MiBP = 0.00001, 0.0025; MnBP = 0.0002, 0.0017, and ΣDBP = 0.0001, 0.0011], respectively). For the girl group, we observed an association between the DEHP metabolites and free T4 according to the crude and creatinine-correction analysis after adjustment for confounders (95% CIs: [crude: MEHP = 0.0004, 0.0031; MEOHP = 0.0003, 0.0024; MEHHP = 0.0002, 0.0018; ΣDEHP = 0.0001, 0.0008]; [creatinine correction: MEHP = 0.0005, 0.0047; MEOHP = 0.0005, 0.0043; MEHHP = 0.0002, 0.0028; ΣDEHP = 0.0002, 0.0014], respectively). MiBP, one metabolite of DBP was associated with free T4 in the girl group according to the crude analysis after adjustment for confounders (95% CIs: 0.0002, 0.0016). However, after creatinine-corrected analysis, the relationship was not significant. In the girl group, TSH levels revealed to increase with an increase in MnBP according to crude and creatinine-correction analysis (95% CIs, crude: 0.0013, 0.0044; creatinine correction: 0.0016, 0.0073). 

In the comparison of the three groups that were defined according to the 33rd and 66th percentiles, higher MiBP, DEHP, and DBP concentrations exerted larger effects on free T4 (*p*: MiBP = 0.047, ΣDBP = 0.022, ΣDEHP = 0.038; Table 5) according to crude analysis after confounder adjustment. However, the creatinine analysis revealed that free T3 was related to DEHP metabolite concentrations (*P* = 0.041; Table 6). In the boy group, the DBP and DEHP metabolites were positively associated with free T3 according to crude or creatinine-correction analyses after adjustment for confounders (*P*: crude: MiBP = 0.039, MnBP = 0.028, ΣDBP = 0.010, MEHHP = 0.025; creatinine correction: MnBP = 0.031, ΣDBP = 0.037, MEHHP = 0.002, MEOHP = 0.037, ΣDEHP = 0.007, respectively; Table 5 and Table 6). In the girl group, DBP metabolite concentrations were positively associated with free T4 concentrations according to crude or creatinine analysis after adjustment for confounders (*P*: crude: MnBP = 0.049, ΣDBP = 0.023; creatinine correction: MnBP = 0.034), respectively; Table 5 and Table 6). Higher MEOHP concentration was discovered to have a large effect on free T4 according to creatinine-correction analysis after adjustment for confounders (*p* = 0.020; Table 6).

## 4. Discussion

The present study revealed that the effect of phthalates on thyroid function varies between boys and girls aged 9–10 years. According to our review of relevant literature, the positive associations between DEHP exposure and free T4 in girls and between DBP exposure and free T3 in boys in our present study were seldom reported in previous observational studies.

The effect of urinary phthalate metabolite concentration on children of different ages and from different regions varies. We determined the urinary excretion of 12 phthalate metabolites in Taiwanese children aged 9–10 years. In a previous study, we examined the urinary phthalate concentrations in the same children when they were aged 2 and 5 years old [20]. The geometric means (standard error) of the MEP, MBP (MiBP and MnBP), MBzP, and MEHP concentrations were 39.74 (1.09), 152.92 (1.05), 3.76 (1.01), and 34.60 (2.28) μg/g creatinine when the children were aged 2 years old and 23.07 (1.09), 57.29 (1.05), 3.46 (1.08), and 14.74 (1.06) μg/g creatinine when the children were aged 5 years old, respectively. The urinary phthalate metabolite concentrations in the children when they were aged 9–10 were significantly lower than those when they were 2 and 5 years old, except for MEP. A study reported higher urinary phthalate metabolite concentrations in children than in adults, possibly because of increased dosage per unit body surface area, and immature renal function in young children. [21]. MEP concentration was discovered to be higher in children aged 9–10 years than those aged 5. MEP was previously revealed to be significantly correlated with the use of hair mousse, hair dye, cosmetics, chewing gum, and polyethylene terephthalate [22]. A previous epidemiological study reported that MEP may increase the risk of breast cancer in women [23]. A long-term follow-up of urinary MEP concentrations is necessary. We also compared the phthalate metabolite concentrations with those reported in previous studies conducted on children and adolescents. The geometric median concentration of MEHP in our study was similar to that reported in an American study on children [24]. The sum of DEHP metabolites was considerably lower in our study than in those conducted in Australia [25], Greece [26], Denmark [27], and Japan [28] and other studies conducted in Taiwan after a food scandal [29]; however, the results were similar to those obtained in China [30]. The MnBP concentration in our study was markedly higher than that reported in a Japanese study [28] and mildly lower than that reported in a Chinese study [30]. The sum of DBP metabolite concentrations in our study was similar to that reported in America [24] but lower than that reported in Australia [25], Denmark [27], Germany [21], and Greece [26]. Furthermore, the MEP concentration in our study was higher than that reported in Danish [27] and Chinese [30] studies but lower than that reported in an Australian study [25]. These results can be attributed to regional and age differences in exposure.

We observed a significant positive association between urinary DEHP metabolites and free T4 in the girls by using a generalized linear model with a log link function in a crude and creatinine-correction analysis after adjustment for confounders. After categorizing each exposure into three groups according to their concentration, urinary DEHP metabolites, particularly MEHHP, were positively associated with free T3 in boys in crude and creatinine-correction analysis after cofounder adjustment, although no significant relationship between urinary DEHP metabolite and thyroid function in boys was discovered, as had been determined using the generalized linear model with a log link function. The two statistical methods yielded different results. We inferred that the effects on thyroid function were insensitive to low concentrations of DEHP in boys; however, higher concentrations of phthalates exerted a large effect on thyroid function. DEHP exposure was positively associated with free T4 in crude and creatinine-corrected analyses in girls, as determined using a log link function analysis. However, girls showed only a marginally positive association with DEHP metabolites and free T4 after each categorization into three groups. In girls, higher urinary DEHP, particularly MEOHP, concentrations exerted larger effects on free T4 after creatinine correction at lower urinary concentrations. Thus, there a may be a ceiling effect regarding DEHP effect on free T4 in girls. These results potentially reflect the effects of varying concentrations of phthalates on thyroid function in boys and girls. Thyroid hormones have extremely complex and multifactorial regulations; therefore, the relationships between different concentrations of phthalate metabolites and thyroid hormones are complicated. We compared our results with those of previous studies. Meeker et al. reported a significantly inverse association between MEHP and serum free T4, but not TSH and T3, after adjustments for covariates in adults. By contrast, MEHHP was significantly and positively associated with free T4, but not TSH and T3, in a subgroup of 208 men [15]. In adolescents, Meeker et al. reported a significant and positive association among DEHP secondary metabolites, T3, and TSH [24]. No relationship was discovered between DEHP metabolites and TSH in our study. Wu et al. interviewed the main caregivers of children affected by the 2011 scandal in Taiwan to construct an exposure matrix of DEHP from contaminated foodstuffs and observed that higher DEHP exposure was more significantly associated with decreased serum TSH levels, despite the study’s small sample size [18]. Our findings differed from those of a Danish study of 845 children (age 4–9) that reported inverse relationships between metabolites of DEHP and other phthalates and free and total T3 [27]. The variations in the study population and participant age may account for these differences in the association; in addition, varying exposure levels among populations may be involved. The median (75th percentile) MEHP concentration in our study was 5.07 (8.8) µg/g creatinine, whereas that in the Danish study was 6.8 (11.0) µg/g creatinine. Furthermore, our findings differed from those of a Taiwanese study (participants age <18) that reported no relationships between urinary DEHP metabolites and thyroid hormones. The median MEHP, MEOHP, and MEHHP concentrations in our study were 5.07, 11.27, and 16.9 µg/g creatinine, respectively. However, another Taiwanese study conducted after the 2011 Taiwan food scandal reported MEHP, MEOHP, and MEHHP concentrations of 36.29, 166.48, and 119.00 µg/g creatinine, respectively [29]. Many molecular and animal studies have reported that DEHP is an antagonist of thyroid hormones [8,11,13,14]. However, Gayathri et al. reported that phthalates can increase levels of thyroid hormones and the proliferation of thyroid hormone–induced cells and suggested that phthalates induce thyroid gland hyperactivity [31]. The variations in the study populations and urinary DEHP concentrations may account for these differences in the association of phthalates with thyroid function.

The two statistical methods revealed significant effects of DBP on free T3 in boys. In girls, the relationship between MnBP metabolites and free T4 was significant after categorization into three groups. MiBP and MnBP are metabolites of DBP, which is commonly used as a plasticizer or an additive in adhesives or printing inks and was previously used for cosmetic purposes. We observed that MnBP was positively associated with TSH in girls, as determined using the generalized linear model with a log link function. Our findings differ from those of a Taiwanese study (participants age <18) that reported no relationships between urinary DBP metabolites and thyroid hormones [29]. The median MnBP and MiBP concentrations discovered in our study were 12.04 and 13.04 µg/g creatinine, respectively. However, these concentrations were 172.55 and 92.27 µg/g creatinine, respectively, in a previous Taiwanese study in children [29]. Variations in study population and urinary DBP concentrations may account for these differences in the association. According to our review of relevant literature, this paper is the first to report that DBP may exert gender-specific effects on different thyroid hormones in children.

The strength of this study was its prospective cohort design and utilization of reliable assessment tools. Prospective data collection minimizes the potential for recall bias and differential misclassification and reveals a clearer temporal relationship. The present study did have some limitations. First, the TBPS recruited children from the Taipei metropolitan area only. Second, we collected only one serum sample and one-spot urine sample to measure thyroid hormone and phthalate metabolite levels. Variations in our serum and urine samples might have underestimated the observed correlation [32]. Third, we only discuss the samples with phthalate metabolite concentrations 50% more than the LOQ. We analyzed 12 phthalate metabolites but only six metabolites satisfied this criterion. However, other phthalate metabolites may have also affected thyroid hormones. Fourth, the effects of phthalates on thyroid function as determined using the two statistical methods were not always consistent because only 189 children (92 boys and 97 girls) were enrolled in our study. Finally, the effect of co-exposure and randomness across multiple comparisons are both critical issues in this study. Multiple significance testing could increase the risk of obtaining random ”false positive” results. However, the number of subjects in our study limited further stratified analysis. A larger sample size may yield more consistent and powerful results. 

## 5. Conclusions

Our results revealed that among the different types of phthalates, DEHP and DBP metabolites had the strongest effect on thyroid hormones in children aged 9–10. A positive association was discovered between DEHP exposure and free T4 in girls and between DBP exposure and free T3 in boys. Moreover, changes in thyroid function were less sensitive to exposure to low concentrations of DEHP; however, a higher concentration of DEHP exerted a larger effect on free T3 in boys. These results potentially reflect the different effects of phthalates on thyroid function in boys compared with girls. In our study, serum thyroid hormone levels were within the normal physiological range; however, the disruption of normal thyroid homeostasis in early life has been reported to be critical during development, and even subtle changes may affect child health. Phthalates exert gender-specific effects that play a pivotal role in thyroid functions and development. Additional studies are necessary to confirm and further elucidate the exact mechanisms underlying our findings. 

## Figures and Tables

**Table 1:** Basic demographics of the study population.

	Boys (92)	Girls (97)	Total (189)
**Mother**			
Maternal age while delivery (≥35 years old) (%)	24.30	31.70	27.90
Maternal education ≥college (%)	47.70	55.40	51.40
Occupation (non-housewife) (%)	86.00	82.20	84.10
**Children**			
Birth weight <2500 g (%)	2.56	5.00	3.80
Gestational age (<37 weeks) (%)	3.70	5.00	4.30
Parity <2 (%)	37.40	42.60	39.90
Height (cm) (8 years old) (mean ± SD)	135.75 ± 5.77	135.62 ± 6.09	135.69 ± 5.91
Weight (kg) (mean ± SD)	33.17 ± 7.43	31.35 ± 6.68	32.29 ± 7.12
BMI (kg/m^2^) (mean ± SD)	17.89 ± 3.22	16.93 ± 2.74	17.42 ± 3.02
**Environmental factors**			
ETS exposures (%)	30.20	18.60	24.40
Family income per year >1,500,000 (NT dollars) (%)	15.90	17.80	16.80

ETS: environmental tobacco smoke. NT: New Taiwan dollars.

**Table 2:** Concentrations of urinary phthalate metabolites.

Phthalate Metabolites	LOQ (μg/L)	Percent (%) > LOQ	25th Percentile	Median	75th Percentile	95th Percentile
**Without creatinine correction (μg/L)**						
MMP	0.10	29.20	<LOQ	<LOQ	1.72	12.06
MEP	0.50	96.30	16.83	30.49	69.60	156.90
MiBP	0.50	92.70	5.96	15.46	31.21	78.36
MnBP	0.50	90.40	5.64	15.60	35.72	96.84
MBzP	0.05	47.00	<LOQ	<LOQ	1.43	7.28
MEHP	0.50	86.30	1.31	4.44	9.38	30.95
MEHHP	0.05	100.00	8.03	17.21	33.37	69.97
MEOHP	0.05	100.00	5.36	11.06	21.93	49.18
MCHP	0.50	0.90	<LOQ	<LOQ	<LOQ	<LOQ
MCPP	0.50	1.80	<LOQ	<LOQ	<LOQ	<LOQ
MOP	0.50	0.00	<LOQ	<LOQ	<LOQ	<LOQ
MiNP	0.50	3.20	<LOQ	<LOQ	<LOQ	<LOQ
**Creatinine-corrected concentration (μg/g creatinine) ^a^**						
MMP			<LOQ	<LOQ	2.34	15.08
MEP			18.60	34.82	71.55	153.74
MiBP			7.07	16.57	28.01	62.06
MnBP			6.44	17.17	31.99	75.79
MBzP			<LOQ	<LOQ	1.53	5.04
MEHP			1.86	4.40	8.80	26.28
MEHHP			10.03	20.45	30.91	53.44
MEOHP			6.87	12.84	20.51	34.61
MCHP			<LOQ	<LOQ	<LOQ	<LOQ
MCPP			<LOQ	<LOQ	<LOQ	<LOQ
MOP			<LOQ	<LOQ	<LOQ	<LOQ
MiNP			<LOQ	<LOQ	<LOQ	<LOQ

^a^: The samples with phthalate metabolite concentrations higher than the LOQ by more than 50% were listed.

**Table 3:** Serum concentrations of thyroid hormones.

Samples	Free T4 (ng/dL)	Total T4 (μg/dL)	Free T3 (ng/dL)	Total T3 (μg/dL)	TSH (miu/mL)
**all**					
(*n* = 189)					
mean ± SD	1.39 ± 0.14	8.72 ± 1.35	4.06 ± 0.52	151.83 ± 24.00	2.53 ± 1.23
median	1.38	8.60	4.09	151.65	2.33
**boys**					
(*n* = 92)					
mean ± SD	1.39 ± 0.14	8.85 ± 1.39	4.06 ± 0.46	151.17 ± 24.61	2.63 ± 1.26
median	1.40	8.96	4.13	150.30	2.42
**girls**					
(*n* = 97)					
mean ± SD	1.38 ± 0.14	8.57 ± 1.30	4.06 ± 0.59	152.55 ± 23.43	2.45 ± 1.20
median	1.36	8.43	4.05	153.4	2.22

**Table 4:** Regression coefficientsβ (95% CI) for log-serum thyroid hormones according to log-urine phthalate metabolite concentrations ^a^.

Crude Analysis				Creatinine-Corrected Analysis		
	All	Boys	Girls	All	Boys	Girls
**Free T4**						
MEP	0.00001	−0.00001	0.00001	−0.00001	−0.0001	0.00001
	(−0.0001, 0.0001)	(−0.0004, 0.0004)	(−0.0001, 0.0002)	(−0.0001, 0.0001)	(−0.0004, 0.0003)	(−0.0001, 0.0001)
MiBP	0.0004	−0.0003	0.0009 *	0.0004	−0.0004	0.0007
	(−0.0002, 0.0009)	(−0.0011, 0.0006)	(0.0002, 0.0016)	(−0.0003, 0.0001)	(−0.0016, 0.0008)	(−0.0001, 0.0015)
MnBP	0.0003	0.0004	0.0003	0.0004	0.0004	0.0003
	(−0.00001, 0.0007)	(−0.0002, 0.0010)	(−0.0001, 0.0007)	(−0.0002, 0.0009)	(−0.0004, 0.0011)	(−0.0004, 0.0010)
ΣDBP	0.0002	0.0001	0.0003	0.0002	0.0001	0.0003
	(−0.00001, 0.0004)	(−0.0003, 0.0005)	(−0.00001, 0.0006)	(−0.0001, 0.0006)	(−0.0004, 0.0006)	(−0.0001, 0.0008)
MEHP	−0.00001	−0.0001	0.0018 *	−0.00001	−0.0001	0.0026 *
	(−0.0002, 0.0002)	(−0.0002, 0.0001)	(0.0004, 0.0031)	(−0.0004, 0.0003)	(−0.0005, 0.0002)	(0.0005, 0.0047)
MEHHP	0.0001	−0.0001	0.0010	0.0001	−0.0002	0.0015 *
	(−0.0002, 0.0004)	(−0.0005, 0.0002)	(0.0002, 0.0018)	(−0.0004, 0.0006)	(−0.0008, 0.0001)	(0.0002, 0.0028)
MEOHP	0.0001	−0.0002	0.0014 *	0.0002	−0.0004	0.0024 *
	(−0.0003, 0.0006)	(−0.0007, 0.0003)	(0.0003, 0.0024)	(−0.0006, 0.0010)	(−0.0013, 0.0006)	(0.0005, 0.0043)
ΣDEHP	0.00001	−0.00001	0.0005 *	0.00001	−0.0001	0.0008 *
	(−0.0001, 0.0001)	(−0.0001, 0.0001)	(0.0001, 0.0008)	(−0.0002, 0.0002)	(−0.0003, 0.0001)	(0.0002, 0.0014)
**Total T4**						
MEP	−0.00001	−0.0001	−0.00001	−0.00001	−0.0001	0.00001
	(−0.0002, 0.0002)	(−0.0006, 0.0005)	(−0.0002, 0.0002)	(−0.0001, 0.0001)	(−0.0006, 0.0004)	(−0.0001, 0.0001)
MiBP	0.0007	0.0007	0.0005	0.0010 *	0.0012	0.0009
	(−0.0001, 0.0015)	(−0.0005, 0.0020)	(−0.0006, 0.0016)	(0.00001, 0.0019)	(−0.0007, 0.0030)	(−0.0003, 0.0020)
MnBP	0.0003	0.0005	0.0001	0.0004	0.0004	0.0002
	(−0.0002, 0.0008)	(−0.0004, 0.0014)	(−0.0006, 0.0007)	(−0.0004, 0.0012)	(−0.0007, 0.0016)	(−0.0009, 0.0012)
ΣDBP	0.0003	0.0003	0.0001	0.0004	0.0004	0.0003
	(−0.0001, 0.0006)	(−0.0002, 0.0009)	(−0.0003, 0.0006)	(−0.0001 (0.0009)	(−0.0004, 0.0011)	(−0.0003, 0.0010)
MEHP	0.00001	0.00001	0.0002	0.0001	0.00001	0.0006
	(−0.0002, 0.0003)	(−0.0003, 0.0003)	(−0.0020, 0.0024)	(−0.0004, 0.0006)	(−0.0005, 0.0005)	(−0.0027, 0.0039)
MEHHP	0.0001	0.00001	−0.0002	0.0002	0.0001	−0.0002
	(−0.0003, 0.0005)	(−0.0005, 0.0005)	(−0.0015, 0.0011)	(−0.0006, 0.0010)	(−0.0007, 0.0010)	(−0.0022, 0.0018)
MEOHP	0.0001	0.0001	−0.0001	0.0003	0.0002	−0.0002
	(−0.0005, 0.0008)	(−0.0007, 0.0008)	(−0.0018, 0.0016)	(−0.0008, 0.0015)	(−0.0011, 0.0015)	(−0.0032, 0.0027)
ΣDEHP	0.00001	0.00001	−0.00001	0.0002	0.00001	0.00001
	(−0.0001, 0.0002)	(−0.0001, 0.0001)	(−0.0006, 0.0005)	(−0.0002, 0.0003)	(−0.0002, 0.0003)	(−0.0010, 0.0009)
**Free T3**						
MEP	0.00001	0.0002	0.00001	0.00001	0.0002	0.00001
	(−0.0001, 0.0002)	(−0.0002, 0.0006)	(−0.0002, 0.0002)	(−0.0001, 0.0001)	(−0.0002, 0.0006)	(−0.0001, 0.0001)
MiBP	0.0005	0.0009 *	0.0003	0.0003	0.0013 *	0.0001
	(−0.0002, 0.0011)	(−0.00001, 0.0017)	(−0.0008, 0.0014)	(−0.0005, 0.0012)	(0.00001, 0.0025)	(−0.0011, 0.0013)
MnBP	0.0003	0.0006 *	0.0002	0.0005	0.0009 *	0.0003
	(−0.0001, 0.0008)	(0.00001, 0.0013)	(−0.0005, 0.0009)	(0.0001, 0.0012)	(0.0002, 0.0017)	(−0.0008, 0.0014)
ΣDBP	0.0002	0.0004 *	0.0002	0.0003	0.0006	0.0001
	(−0.0001, 0.0005)	(0.00001, 0.0008)	(−0.0003, 0.0006)	(−0.0001, 0.0007)	(0.0001, 0.0011)	(−0.0006, 0.0008)
MEHP	0.0001	0.0001	0.0004	0.00001	0.0001	0.0004
	(−0.0001, 0.0003)	(−0.0001, 0.0002)	(−0.0018, 0.0025)	(−0.0003, 0.0005)	(−0.0002, 0.0005)	(−0.0028, 0.0037)
MEHHP	0.0002	0.0002	0.0003	0.0004	0.0005	0.0001
	(−0.0001, 0.0006)	(−0.0001, 0.0005)	(−0.0010, 0.0015)	(−0.0002, 0.0010)	(−0.0001, 0.0010)	(−0.0019, 0.0020)
MEOHP	0.0003	0.0003	0.0004	0.0005	0.0006	0.0003
	(−0.0003, 0.0008)	(−0.0002, 0.0008)	(−0.0013, 0.0020)	(−0.0004, 0.0015)	(−0.0002, 0.0015)	(−0.0026, 0.0032)
ΣDEHP	0.00001	0.00001	0.0001	0.0001	0.0001	0.0001
	(−0.0001, 0.0002)	(−0.00001, 0.0001)	(−0.0004, 0.0007)	(−0.0001, 0.0003)	(−0.0001, 0.0003)	(−0.0009, 0.0010)
**Total T3**						
MEP	0.00001	−0.00001	0.00001	0.00001	0.00001	0.00001
	(−0.0002, 0.0002)	(−0.0006, 0.0005)	(−0.0002, 0.0002)	(−0.0001, 0.0001)	(−0.0005, 0.0005)	(−0.0001, 0.0001)
MiBP	−0.0002	0.0002	−0.0005	−0.0003	0.0002	−0.0004
	(−0.0010, 0.0007)	(−0.0010, 0.0015)	(−0.0017, 0.0007)	(−0.0013, 0.0008)	(−0.0017, 0.0020)	(−0.0018, 0.0009)
MnBP	−0.0001	−0.0001	−0.0002	−0.001	−0.0001	−0.0002
	(−0.0007, 0.0005)	(−0.0010, 0.0009)	(−0.0009, 0.0006)	(−0.0010, 0.0007)	(−0.0012, 0.0011)	(−0.0014, 0.0011)
ΣDBP	−0.0001	0.00001	−0.0002	−0.0001	−0.00001	−0.0002
	(−0.0005, 0.0003)	(−0.0006, 0.0006)	(−0.0007, 0.0004)	(−0.0006, 0.0004)	(−0.0008, 0.0007)	(−0.0010, 0.0006)
MEHP	0.00001	0.00001	−0.0014	0.00001	0.0001	−0.0023
	(−0.0002, 0.0003)	(−0.0002, 0.0003)	(−0.0039, 0.0010)	(−0.0005, 0.0005)	(−0.0004, 0.0006)	(−0.0059, 0.0014)
MEHHP	−0.0001	0.0001	−0.0012	−0.0001	0.0002	−0.0021
	(−0.0006, 0.0004)	(−0.0004, 0.0006)	(−0.0026, 0.0002)	(−0.0010, 0.0007)	(−0.0006, 0.0011)	(−0.0042, 0.0001)
MEOHP	−0.0001	0.0002	−0.0014	−0.0002	0.0004	−0.0027
	(−0.0009, 0.0006)	(−0.0006, 0.0009)	(−0.0033, 0.0004)	(−0.0014, 0.0011)	(−0.0009, 0.0016)	(−0.0059, 0.0004)
ΣDEHP	−0.00001	0.00001	−0.0005	−0.00001	0.0001	−0.0009
	(−0.0002, 0.0001)	(−0.0001, 0.0002)	(−0.0011, 0.0001)	(−0.0003, 0.0003)	(−0.0002, 0.0003)	(−0.0019, 0.0001)
**TSH**						
MEP	0.0001	−0.0008	0.0002	0.00001	−0.0005	0.0001
	(−0.0004, 0.0006)	(−0.0029, 0.0013)	(−0.0003, 0.0007)	(−0.0003, 0.0004)	(−0.0023, 0.0014)	(−0.0003, 0.0004)
MiBP	0.0001	−0.0007	0.0009	−0.0019	−0.0022	−0.0014
	(−0.0027, 0.0029)	(−0.0056, 0.0041)	(−0.0026, 0.0043)	(−0.0056, 0.0017)	(−0.0092, 0.0048)	(−0.0058, 0.0029)
MnBP	0.0016	−0.0013	0.0028 *	0.0014	−0.0019	0.0045 *
	(0.0001, 0.0031)	(−0.0051, 0.0024)	(0.0013, 0.0044)	(−0.0011, 0.0039)	(−0.0063, 0.0024)	(0.0016, 0.0073)
ΣDBP	0.0008	−0.0007	0.0018 *	0.0001	−0.0013	0.0013
	(−0.0004, 0.0020)	(−0.0029, 0.0016)	(0.0005, 0.0031)	(−0.0016, 0.0018)	(−0.0042, 0.0017)	(−0.0008, 0.0035)
MEHP	0.0004	0.0003	0.0021	0.0007	0.0007	0.0019
	(−0.0002, 0.0010)	(−0.0003, 0.0010)	(−0.0047, 0.0089)	(−0.0005, 0.0019)	(−0.0006, 0.0019)	(−0.0092, 0.0130)
MEHHP	0.0007	0.0006	0.0017	0.0011	0.0010	0.0007
	(−0.0005, 0.0019)	(−0.0008, 0.0019)	(−0.0022, 0.0057)	(−0.0013, 0.0034)	(−0.0017, 0.0036)	(−0.0061, 0.0075)
MEOHP	0.0012	0.0009	0.0028	0.0020	0.0017	0.0024
	(−0.0005, 0.0029)	(−0.0011, 0.0030)	(−0.0022, 0.0077)	(−0.0014, 0.0053)	(−0.0021, 0.0055)	(−0.0074, 0.0123)
ΣDEHP	0.0002	0.0002	0.0008	0.0004	0.0003	0.0006
	(−0.0001, 0.0005)	(−0.0002, 0.0005)	(−0.0009, 0.0025)	(−0.0003, 0.0010)	(−0.0004, 0.0010)	(−0.0026, 0.0038)

^a^ Adjusted for maternal age ≥35 years old, maternal education ≥college, family income per year >1,500,000 (NT dollars), and birth weight ≥2500 g. Abbreviation: ΣDBP: sum of concentrations of all measured DBP metabolites (MiBP and MnBP); ΣDEHP, sum of concentrations of all measured DEHP metabolites (MEHP, MEHHP, and MEOHP). * *p* < 0.05.

**Table 5:** Association of thyroid hormones and urine phthalate metabolites in three groups comparison (without creatinine correction) ^a^.

	Concentrations (μg/L)	Free T4 (ng/dL)	*p*	Total T4 (μg/dL)	*p*	Free T3 (ng/dL)	*p*	Total T3 (μg/dL)	*p*	TSH (miu/mL)	*p*
**all**											
MEP			0.533		0.825		0.166		0.492		0.810
	<20.07	1.37		8.69		3.98		153.47		2.58	
	20.07–51.41	1.39		8.62		4.15		150.72		2.49	
	>51.41	1.39		8.77		4.10		150.98		2.53	
MiBP			0.047 *		0.167		0.236		0.178		0.260
	<7.96	1.36		8.58		4.07		156.74		2.77	
	7.96–23.12	1.38		8.64		3.99		148.14		2.29	
	>23.12	1.40		8.86		4.16		150.22		2.53	
MnBP			0.060		0.338		0.322		0.310		0.533
	<8.45	1.36		8.55		4.07		154.65		2.44	
	8.45–15.65	1.38		8.82		4.01		151.03		2.59	
	>15.65	1.40		8.72		4.15		149.57		2.57	
ΣDBP			0.022 *		0.138		0.273		0.140		0.919
	<15.86	1.36		8.59		4.07		156.15		2.62	
	15.86–52.78	1.38		8.64		4.00		150.01		2.39	
	>52.78	1.41		8.85		4.16		149.04		2.59	
MEHP			0.166		0.296		0.153		0.865		0.441
	<2.09	1.35		8.42		3.97		150.49		2.75	
	2.09–7.67	1.41		9.00		4.15		155.16		2.28	
	>7.67	1.39		8.67		4.10		149.53		2.58	
MEHHP			0.078		0.187		0.071		0.507		0.915
	<9.71	1.36		8.63		4.02		155.54		2.58	
	9.71–25.58	1.38		8.52		4.01		147.01		2.42	
	>25.58	1.40		8.93		4.19		152.58		2.60	
MEOHP			0.095		0.228		0.179		0.419		0.778
	<6.35	1.36		8.59		4.02		154.20		2.64	
	6.35–16.77	1.38		8.61		4.07		150.19		2.39	
	>16.77	1.40		8.88		4.14		150.82		2.57	
ΣDEHP			0.038 *		0.101		0.182		0.489		0.821
	<19.09	1.35		8.51		4.04		154.24		2.64	
	19.09–47.87	1.39		8.68		4.03		149.74		2.38	
	>47.87	1.40		8.89		4.16		151.22		2.59	
**boys**											
MEP			0.916		0.234		0.303		0.720		0.226
	<20.07	1.38		8.49		3.98		149.47		2.99	
	20.07–51.41	1.40		8.80		4.18		148.18		2.41	
	>51.41	1.38		9.05		4.12		152.97		2.53	
MiBP			0.421		0.137		0.039 *		0.646		0.131
	<7.96	1.37		8.60		4.02		153.46		3.04	
	7.96–23.12	1.39		8.77		4.06		148.14		2.32	
	>23.12	1.40		9.07		4.22		149.50		2.50	
MnBP			0.679	8.50	0.279	4.02	0.028 *	150.73	0.876	2.73	0.628
	<8.45	1.39		8.50		4.02		150.73		2.73	
	8.45–15.65	1.38		9.01		3.99		149.15		2.65	
	>15.65	1.39		8.86		4.26		151.49		2.52	
ΣDBP			0.440		0.097		0.010 *		0.863		0.186
	<15.86	1.38		8.56		4.00		151.41		3.03	
	15.86–52.78	1.39		8.85		4.02		149.22		2.36	
	>52.78	1.39		9.00		4.26		150.78		2.53	
MEHP			0.777		0.213		0.191		0.498		0.348
	<2.09	1.36		8.22		3.97		144.37		3.03	
	2.09–7.67	1.42		9.29		4.14		155.52		2.32	
	>7.67	1.38		8.74		4.14		149.61		2.65	
MEHHP			0.471		0.092		0.025 *		0.665		0.423
	<9.71	1.38		8.62		4.04		153.22		2.84	
	9.71–25.58	1.38		8.59		3.95		144.25		2.55	
	>25.58	1.40		9.18		4.28		154.97		2.56	
MEOHP			0.643		0.332		0.122		0.888		0.276
	<6.35	1.38		8.60		4.03		152.38		2.87	
	6.35–16.77	1.39		8.81		4.04		147.67		2.58	
	>16.77	1.39		8.97		4.21		151.67		2.49	
ΣDEHP			0.434		0.082		0.061		0.693		0.239
	<19.09	1.36		8.39		4.03		150.28		2.97	
	19.09–47.87	1.40		8.87		4.01		148.54		2.51	
	>47.87	1.39		9.04		4.25		152.87		2.53	
**girls**											
MEP			0.553		0.122		0.309		0.237		0.387
	<20.07	1.36		8.83		3.98		156.46		2.27	
	20.07–51.41	1.38		8.46		4.12		153.02		2.56	
	>51.41	1.39		8.42		4.09		148.50		2.53	
MiBP			0.075		0.917		0.768		0.191		0.888
	<7.96	1.34		8.56		4.13		160.13		2.50	
	7.96–23.12	1.37		8.51		3.92		148.15		2.27	
	>23.12	1.41		8.68		4.11		150.83		2.56	
MnBP			0.049 *		0.795		0.863		0.146		0.178
	<8.45	1.34		8.59		4.10		157.48		2.23	
	8.45–15.65	1.38		8.61		4.02		153.09		2.52	
	>15.65	1.41		8.57		4.04		147.54		2.63	
ΣDBP			0.023 *	8.61	0.928	4.12	0.855	160.19	0.041	2.27	0.224
	<15.86	1.34		8.61		4.12		160.19		2.27	
	15.86–52.78	1.36		8.42		3.98		150.90		2.43	
	>52.78	1.42		8.71		4.06		147.45		2.64	
MEHP			0.160		0.868		0.383		0.418		0.721
	<2.09	1.34		8.56		3.98		154.64		2.56	
	2.09–7.67	1.40		8.64		4.15		154.71		2.24	
	>7.67	1.39		8.58		4.07		149.45		2.50	
MEHHP			0.127		0.892		0.473		0.259		0.459
	<9.71	1.35		8.63		4.01		157.00		2.41	
	9.71–25.58	1.39		8.44		4.09		150.60		2.25	
	>25.58	1.40		8.66		4.09		150.11		2.65	
MEOHP			0.098		0.689		0.540		0.377		0.578
	<6.35	1.35		8.58		4.01		155.51		2.47	
	6.35–16.77	1.37		8.39		4.10		153.06		2.18	
	>16.77	1.41		8.77		4.08		149.95		2.67	
ΣDEHP			0.057		0.753		0.674		0.248		0.554
	<19.09	1.34		8.58		4.04		156.57		2.44	
	19.09–47.87	1.37		8.38		4.05		151.63		2.18	
	>47.87	1.41		8.74		4.08		149.71		2.64	

^a^ Adjusted for maternal age ≥35 years old, maternal education ≥college, family income per year >1,500,000 (NT dollars), and birth weight ≥2500 g. Abbreviation: ΣDBP: sum of concentrations of all measured DBP metabolites ( MiBP, and MnBP); ΣDEHP, sum of concentrations of all measured DEHP metabolites (MEHP, MEHHP, and MEOHP). * *p* < 0.05.

**Table 6:** Association of thyroid hormones and urine phthalate metabolites in three groups comparison (with creatinine correction) ^a^.

	Concentrations (μg/L)	Free T4 (ng/dL)	*p*	Total T4 (μg/dL)	*p*	Free T3 (ng/dL)	*p*	Total T3 (μg/dL)	*p*	TSH (miu/mL)	*p*
all											
MEP			0.907		0.276		0.261		0.629		0.473
	<23.07	1.38		8.58		4.01		151.29		2.70	
	23.07~50.55	1.38		8.58		4.12		150.50		2.34	
	50.55<	1.39		8.92		4.10		153.30		2.57	
MiBP			0.289		0.431		0.528		0.128		0.248
	<9.49	1.37		8.65		4.07		156.52		2.71	
	9.49~24.19	1.37		8.57		4.05		149.26		2.40	
	24.19<	1.40		8.87		4.11		149.49		2.49	
MnBP			0.057		0.280		0.382		0.289		0.806
	<9.52	1.36		8.62		4.10		156.50		2.56	
	9.52~26.47	1.38		8.56		3.95		146.84		2.41	
	26.47<	1.41		8.90		4.17		151.68		2.63	
ΣDBP			0.130		0.174		0.699		0.220		0.620
	<23.24	1.36		8.51		4.09		155.54		2.62	
	23.24~52.28	1.39		8.75		4.03		149.98		2.47	
	52.28<	1.40		8.82		4.11		149.74		2.52	
MEHP			0.337		0.714		0.205		0.686		0.289
	<2.74	1.36		8.55		4.01		151.07		2.78	
	2.74~6.42	1.40		8.91		4.09		154.37		2.30	
	6.42<	1.38		8.63		4.13		149.82		2.53	
MEHHP			0.146		0.281		0.039		0.626		0.829
	<13.04	1.35		8.42		3.98		152.34		2.60	
	13.04~25.93	1.40		8.94		4.08		151.91		2.45	
	25.93<	1.39		8.74		4.17		150.93		2.55	
MEOHP			0.098		0.326		0.127		0.462		0.214
	<9.15	1.35		8.43		4.02		153.75		2.69	
	9.15~17.95	1.39		8.94		4.06		150.51		2.47	
	17.95<	1.40		8.72		4.15		150.90		2.44	
ΣDEHP			0.175	8.36	0.168	3.97	0.041 *	151.62	0.885	2.65	0.339
	<25.34	1.35		8.36		3.97		151.62		2.65	
	25.34~49.92	1.41		8.98		4.11		152.34		2.50	
	49.92<	1.38		8.75		4.15		151.21		2.45	
**boys**											
MEP			0.434		0.138		0.201		0.141		0.175
	<23.07	1.40		8.53		4.00		146.99		2.92	
	23.07~50.55	1.37		8.67		4.13		146.78		2.39	
	50.55<	1.39		9.21		4.16		157.06		2.52	
MiBP			0.785		0.223		0.087		0.722		0.187
	<9.49	1.38		8.58		3.98		151.79		2.96	
	9.49~24.19	1.40		8.87		4.18		150.36		2.33	
	24.19<	1.38		9.03		4.15		148.80		2.55	
MnBP			0.652		0.214		0.031 *		0.885		0.280
	<9.52	1.38		8.62		4.03		152.36		2.79	
	9.52~26.47	1.37		8.65		3.96		143.43		2.65	
	26.47<	1.40		9.12		4.27		154.03		2.45	
ΣDBP			0.828		0.151		0.037 *		0.960		0.203
	<23.24	1.38		8.50		3.99		150.23		2.85	
	23.24~52.28	1.40		9.05		4.13		152.76		2.56	
	52.28<	1.39		8.95		4.20		148.43		2.42	
MEHP			0.652		0.691		0.262		0.801		0.294
	<2.74	1.37		8.49		4.03		147.54		2.93	
	2.74~6.42	1.43		9.24		4.07		153.57		2.41	
	6.42<	1.36		8.70		4.17		150.13		2.57	
MEHHP			0.657		0.181		0.002 *		0.346		0.249
	<13.04	1.37		8.39		3.90		145.66		2.85	
	13.04~25.93	1.40		9.02		4.10		152.47		2.56	
	25.93<	1.39		8.98		4.28		152.83		2.49	
MEOHP			0.816		0.572		0.037 *		0.866		0.099
	<9.15	1.39		8.57		3.98		149.23		2.98	
	9.15~17.95	1.38		8.93		4.07		149.92		2.43	
	17.95<	1.39		8.91		4.24		152.18		2.49	
ΣDEHP			0.843		0.273		0.007 *		0.401		0.108
	<25.34	1.37		8.38		3.90		146.20		2.93	
	25.34~49.92	1.42		9.08		4.13		151.74		2.55	
	49.92<	1.37		8.91		4.23		152.87		2.44	
**girls**											
MEP			0.451		0.723		0.612		0.364		0.585
	<23.07	1.35		8.63		4.01		156.51		2.44	
	23.07~50.55	1.38		8.52		4.11		153.19		2.30	
	50.55<	1.39		8.63		4.04		149.65		2.61	
MiBP			0.090		0.722		0.861		0.075		0.945
	<9.49	1.35		8.73		4.19		162.26		2.41	
	9.49~24.19	1.34		8.28		3.92		148.20		2.47	
	24.19<	1.42		8.75		4.08		150.00		2.45	
MnBP			0.034*		0.862		0.663		0.074		0.113
	<9.52	1.33		8.61		4.18		161.06		2.30	
	9.52~26.47	1.38		8.50		3.95		149.37		2.24	
	26.47<	1.41		8.67		4.07		149.25		2.81	
ΣDBP			0.060		0.775		0.450		0.108		0.384
	<23.24	1.33		8.52		4.22		162.42		2.32	
	23.24~52.28	1.38		8.51		3.95		147.69		2.38	
	52.28<	1.41		8.72		4.04		150.80		2.60	
MEHP			0.123		0.805		0.416		0.420		0.530
	<2.74	1.35		8.60		3.99		154.18		2.65	
	2.74~6.42	1.38		8.60		4.10		155.11		2.20	
	6.42<	1.40		8.57		4.10		149.50		2.48	
MEHHP			0.145		0.998		0.727		0.118		0.482
	<13.04	1.34		8.45		4.04		158.05		2.39	
	13.04~25.93	1.41		8.83		4.05		151.24		2.32	
	25.93<	1.39		8.53		4.08		149.35		2.60	
MEOHP			0.020*		0.582		0.709		0.192		0.836
	<9.15	1.32		8.29		4.05		157.86		2.43	
	9.15~17.95	1.40		8.95		4.06		151.13		2.51	
	17.95<	1.41		8.56		4.07		149.77		2.40	
ΣDEHP			0.060		0.573		0.573		0.286		0.944
	<25.34	1.33		8.34		4.02		156.09		2.42	
	25.34~49.92	1.41		8.87		4.08		152.99		2.45	
	49.92<	1.40		8.60		4.08		149.66		2.47	

^a^ Adjusted for maternal age ≥35 years old, maternal education ≥ college, family income per year >1,500,000 (NT dollars), and birth weight ≥2500 g. Abbreviation: ΣDBP: sum of concentrations of all measured DBP metabolites ( MiBP, and MnBP); ΣDEHP, sum of concentrations of all measured DEHP metabolites (MEHP, MEHHP, and MEOHP). * *p* < 0.05.

## Supplementary Materials

The following are available online at www.mdpi.com/1660-4601/14/2/123/s1, Table S1: Regression coefficients β (95% CI) for log-serum thyroid hormones according to log-urine phthalate metabolite concentrations, Table S2: Association of thyroid hormones and urine phthalate metabolites in three groups comparison (without creatinine correction), Table S3: Association of thyroid hormones and urine phthalate metabolites in three groups comparison (the correction of creatinine).

## Abbreviations

The following abbreviations are used in this manuscript:

BZBP	Benzylbutyl phthalate metabolite
DBP	Dibutyl phthalate
DCHP	Dicyclohexyl phthalate metabolite
DEP	Diethyl phthalate
DEHP	Di-2-ethylhexyl phthalate
DiNP	Diisononyl phthalate
DMP	Dimethyl phthalate
DOP	Di-n-octyl phthalate
LOQ	Limit of quantification
MBzP	Mono-benzyl phthalate
MCPP	Mono-3-carboxypropyl phthalate
MCHP	Monocyclohexyl phthalate
MEHHP	Mono-2-ethyl-5-hydroxyhexyl phthalate
MEHP	Mono-(2-ethylhexyl) phthalate
MEOHP	Mono-2-ethyl-5-oxohexyl phthalate
MEP	Monoethyl phthalate
MiBP	Mono-isobutyl phthalate
MnBP	Mono-n-butyl phthalate
MiNP	Mono-isononyl phthalate
MMP	Monomethyl phthalate
MOP	Mono-n-octyl phthalate
TSH	Thyroid-stimulating hormone
T3	Triiodothyronine
T4	Thyroxine
UPLC –MS/MS	Ultrahigh-performance liquid chromatography (UPLC)–tandem mass spectrometry
