# Supplementary material for: Effects of Gender on the Association of Urinary Phthalate Metabolites with Thyroid Hormones in Children: A Prospective Cohort Study in Taiwan
Source: Int J Environ Res Public Health. 2017 Jan 29;14(2):123. doi: 10.3390/ijerph14020123 (PMC5334677; doi:10.3390/ijerph14020123)
Supplement: Supplementary file 1 [file ijerph-14-00123-s001.pdf]

Supplementary Materials: Effects of Gender on the Association of Urinary Phthalate Metabolites with Thyroid Hormones in Children: A Prospective Cohort Study in Taiwan

Te-I Weng, Mei-Huei Chen, Guang-Wen Lien, Pai-Shan Chen, Jasper Chia-Cheng Lin, Cheng-Chung Fang and Pau-Chung Chen

Table S1. Regression coefficients β (95% CI) for log-serum thyroid hormones according to log-urine phthalate metabolite concentrations.

	Crude Analysis			Creatinine-Corrected Analysis		
	**All**	**Boys**	**Girls**	**All**	**Boys**	**Girls**
**Free T4**						
MEP	0.00001<br>(–0.0001, 0.0001)	0.00001<br>(–0.0003, 0.0004)	0.00001<br>(–0.0001, 0.0002)	–0.00001<br>(–0.0001, 0.0001)	–0.00001<br>(–0.0003, 0.0003)	–0.00001<br>(–0.0001, 0.0001)
MiBP	0.0004<br>(–0.0001, 0.0009)	–0.0003<br>(–0.0011, 0.0005)	0.0009 *<br>(0.0003, 0.0016)	0.0004<br>(–0.0002, 0.0011)	–0.0003<br>(–0.0015, 0.0009)	0.0008 *<br>(0.0001, 0.0015)
MnBP	0.0003<br>(–0.00001, 0.0007)	0.0003<br>(–0.0003, 0.0009)	0.0003<br>(–0.0001, 0.0008)	0.0004<br>(–0.0001, 0.0009)	0.0004<br>(–0.0004, 0.0011)	0.0004<br>(–0.0003, 0.0011)
ΣDBP	0.0002<br>(–0.00001, 0.0004)	0.0001<br>(–0.0003, 0.0004)	0.0003<br>(0.00001, 0.0006)	0.0003<br>(–0.0001, 0.0006)	0.0001<br>(–0.0004, 0.0006)	0.0004<br>(–0.00001, 0.0008)
MEHP	–0.00001<br>(–0.0002, 0.0002)	–0.0001<br>(–0.0002, 0.0001)	0.0018 *<br>(0.0005, 0.0031)	–0.00001<br>(–0.0004, 0.0003)	–0.0001<br>(–0.0005, 0.0003	0.0027 *<br>(0.0006, 0.0048)
MEHHP	0.0001<br>(–0.0002, 0.0003)	–0.0001<br>(–0.0005, 0.0002)	0.0010 *<br>(0.0003, 0.0018)	0.0001<br>(–0.0004, 0.0006)	0.0002<br>(–0.0008, 0.0004)	0.0015 *<br>(0.0003, 0.0028)
MEOHP	0.0001<br>(–0.0003, 0.0006)	–0.0002<br>(–0.0007, 0.0004)	0.0014 **<br>(0.0004, 0.0025)	0.0002<br>(–0.0006, 0.0010)	–0.0003<br>(–0.0012, 0.0007)	0.0025 *<br>(0.0006, 0.0043)
ΣDEHP	0.00001<br>(–0.0001, 0.0001)	0.00001<br>(–0.0001, 0.0001)	0.0005 *<br>(0.0001, 0.0008)	0.00001<br>(–0.0002, 0.0002)	–0.0001<br>(–0.0002, 0.0001)	0.0008 *<br>(0.0002, 0.0014)

Table S1. *Cont.*

	Crude Analysis			Creatinine-Corrected Analysis		
	**All**	**Boys**	**Girls**	**All**	**Boys**	**Girls**
**Total T4**						
MEP	0.00001 (–0.0002, 0.0002)	0.0001 (–0.0004, 0.0007)	–0.00001 (–0.0002, 0.0002)	0.00001 (–0.0001, 0.0001)	0.0001 (–0.0004, 0.0006)	0.00001 (–0.0001, 0.0001)
MiBP	0.0007 (–0.0001,.0015)	0.0008 (–0.0005, 0.0020)	0.0007 (–0.0003, 0.0018)	0.0010 * (0.0001, 0.0020)	0.0012 (–0.0006, 0.0030)	0.0011 (–0.00001, 0.0022)
MnBP	0.0003 (–0.0002, 0.0008)	0.0005 (–0.0004, 0.0014)	0.0002 (–0.0004, 0.0009)	0.0005 (–0.0003, 0.0012)	0.0005 (–0.0007, 0.0016)	0.0004 (–0.0006, 0.0015)
ΣDBP	0.0003 (–0.0001, 0.0006)	0.0003 (–0.0002, 0.0009)	0.0002 (–0.0002, 0.0007)	0.0004 (–0.0001, 0.0009)	0.0004 (–0.0003, 0.0012)	0.0005 (–0.0001, 0.0011)
MEHP	0.00001 (–0.0002, 0.0003)	0.00001 (–0.0003, 0.0003)	0.0005 (–0.0016, 0.0026)	0.0001 (–0.0004, 0.0006)	0.00001 (–0.0005, 0.0006)	0.0010 (–0.0023, 0.0043)
MEHHP	0.0001 (–0.0004, 0.0005)	0.0001 (–0.0004, 0.0006)	–0.00001 (–0.0013, 0.0013)	0.0003 (–0.0005, 0.0010)	0.0002 (–0.0006, 0.0011)	0.0001 (–0.0019, 0.0021)
MEOHP	0.0002 (–0.0005, 0.0008)	0.0001 (–0.0006, 0.0009)	0.0001 (–0.0016, 0.0018)	0.0005 (–0.0007, 0.0016)	0.0004 (–0.0009, 0.0017)	0.0003 (–0.0026, 0.0032)
ΣDEHP	0.00001 (–0.0001, 0.0002)	0.00001 (–0.0001, 0.0002)	0.00001 (–0.0005, 0.0006)	0.0001 (–0.0002, 0.0003)	0.0001 (–0.0002, 0.0003)	0.0001 (–0.0008, 0.0011)

Table S1. *Cont.*

	Crude Analysis			Creatinine-Corrected Analysis		
	**All**	**Boys**	**Girls**	**All**	**Boys**	**Girls**
**Free T3**						
MEP	0.00001 (–0.0001, 0.0002)	0.0002 (–0.0002, 0.0006)	0.00001 (–0.0002, 0.0002)	0.00001 (–0.0001, 0.0001)	0.0002 (–0.0001, 0.0006)	0.00001 (–0.0001, 0.0001)
MiBP	0.0004 (–0.0003, 0.0011)	0.0008 (–0.00001, 0.0016)	0.0001 (–0.0009, 0.0012)	0.0002 (–0.0006, 0.0010)	0.0011 (–0.0001, 0.0023)	–0.0001 (–0.0013, 0.0010)
MnBP	0.0003 (–0.0002, 0.0007)	0.0006 * (0.00001, 0.0012)	0.0001 (–0.0006, 0.0007)	0.0004 (–0.0002, 0.0011)	0.0009 * (0.0002, 0.0017)	0.00001 (–0.0010, 0.0011)
ΣDBP	0.0002 (–0.0001, 0.0005)	0.0004 * (0.00001, 0.0008)	0.0001 (–0.0004, 0.0005)	0.0002 (–0.0002, 0.0006)	0.0006 * (0.0001, 0.0011)	–0.00001 (–0.0007, 0.0006)
MEHP	0.0001 (–0.0001, 0.0003)	0.0001 (–0.0001, 0.0002)	0.0002 (–0.0019, 0.0024)	0.0001 (–0.0003, 0.0005)	0.0001 (–0.0002, 0.0005)	0.0002 (–0.0030, 0.0035)
MEHHP	0.0002 (–0.0001, 0.0006)	0.0002 (–0.0001, 0.0005)	0.0002 (–0.0011, 0.0014)	0.0004 (–0.0002, 0.0011)	0.0005 (–0.0001, 0.0010)	–0.0001 (–0.0020, 0.0019)
MEOHP	0.0003 (–0.0003, 0.0008)	0.0003 (–0.0002, 0.0008)	0.0002 (–0.0014, 0.0019)	0.0006 (–0.0004, 0.0015)	0.0006 (–0.0002, 0.0015)	0.00001 (–0.0028, 0.0029)
ΣDEHP	0.00001 (–0.0001, 0.0002)	0.00001 (–0.00001, 0.0001)	0.0001 (–0.0005, 0.0006)	0.0001 (–0.0001, 0.0003)	0.0001 (–0.0001, 0.0003)	0.00001 (–0.0009, 0.0009)

Table S1. *Cont.*

	Crude Analysis			Creatinine-Corrected Analysis		
	**All**	**Boys**	**Girls**	**All**	**Boys**	**Girls**
**Total T3**						
MEP	0.00001 (–0.0002, 0.0002)	0.0001 (–0.0004, 0.0006)	0.0001 (–0.0002, 0.0002)	0.00001 (–0.0001, 0.0001)	0.0002 (–0.0003, 0.0006)	0.00001 (–0.0001, 0.0001)
MiBP	–0.0002 (–0.0011, 0.0006)	0.0002 (–0.0011, 0.0014)	–0.0006 (–0.0017, 0.0006)	–0.0003 (–0.0014, 0.0007)	–0.00001 (–0.0018, 0.0018)	–0.0006 (–0.0001, 0.0001)
MnBP	–0.0001 (–0.0007, 0.0004)	–0.00001 (–0.0010, 0.0009)	–0.0002 (–0.0009, 0.0005)	–0.0002 (–0.0010, 0.0006)	–0.0001 (–0.0012, 0.0011)	–0.0003 (–0.0015, 0.0009)
ΣDBP	–0.0001 (–0.0005, 0.0003)	0.00001 (–0.0005, 0.0006)	–0.0002 (–0.0007, 0.0003)	–0.0002 (–0.0007, 0.0004)	–0.00001 (–0.0008, 0.0007)	–0.0003 (–0.0010, 0.0004)
MEHP	0.00001 (–0.0002, 0.0003)	0.00001 (–0.0002, 0.0003)	–0.0014 (–0.0038, 0.0011)	0.00001 (–0.0005, 0.0005)	0.0001 (–0.0004, 0.0006)	–0.0022 (–0.0058, 0.0014)
MEHHP	–0.0001 (–0.0005, 0.0004)	0.0001 (–0.0003, 0.0006)	–0.0012 (–0.0026, 0.0002)	–0.0001 (–0.0010, 0.0007)	0.0003 (–0.0006, 0.0011)	–0.0020 (–0.0041, 0.0001)
MEOHP	–0.0001 (–0.0009, 0.0006)	0.0002 (–0.0006, 0.0009)	–0.0014 (–0.0033, 0.0004)	–0.0001 (–0.0014, 0.0011)	0.0004 (–0.0009, 0.0017)	–0.0025 (–0.0056, 0.0005)
ΣDEHP	–0.00001 (–0.0001, 0.0001)	0.00001 (–0.0001, 0.0002)	–0.0005 (–0.0011, 0.0001)	–0.00001 (–0.0003, 0.0003)	0.0001 (–0.0002, 0.0003)	–0.0009 (–0.0019, 0.0001)

Table S1. *Cont.*

	Crude Analysis			Creatinine-Corrected Analysis		
	**All**	**Boys**	**Girls**	**All**	**Boys**	**Girls**
**TSH**						
MEP	0.0001 (–0.0004, 0.0006)	–0.0008 (–0.0029, 0.0013)	0.0002 (–0.0003, 0.0007)	0.00001 (–0.0003, 0.0004)	–0.0004 (–0.0022, 0.0014)	0.0001 (–0.0002, 0.0004)
MiBP	0.00001 (–0.0027, 0.0028)	–0.0013 (–0.0060, 0.0034)	0.0011 (–0.0023, 0.0044)	–0.0017 (–0.0053, 0.0019)	–0.0026 (–0.0094, 0.0042)	–0.0010 (–0.0051, 0.0031)
MnBP	0.0016 * (0.0001, 0.0032)	–0.0016 (–0.0053, 0.0021)	0.0027 * (0.0014, 0.0041)	0.0015 (–0.0010, 0.0040)	–0.0021 (–0.0064, 0.0023)	0.0042 * (0.0016, 0.0067)
ΣDBP	0.0008 (–0.0004, 0.0019)	–0.0009 (–0.0031, 0.0013)	0.0017 (0.0006, 0.0029)	0.0002 (–0.0015, 0.0019)	–0.0014 (–0.0043, 0.0015)	0.0013 (–0.0006, 0.0033)
MEHP	0.0003 (–0.0003, 0.0009)	0.0003 (–0.0003, 0.0009)	0.0022 (–0.0044, 0.0088)	0.0007 (–0.0005, 0.0018)	0.0006 (–0.0006, 0.0018)	0.0022 (–0.0085, 0.0130)
MEHHP	0.0006 (–0.0006, 0.0018)	0.0004 (–0.0010, 0.0018)	0.0019 (–0.0020, 0.0057)	0.0010 (–0.0013, 0.0033)	0.0008 (–0.0017, 0.0034)	0.0012 (–0.0055, 0.0079)
MEOHP	0.0011 (–0.0006, 0.0028)	0.0008 (–0.0012, 0.0028)	0.0029 (–0.0019, 0.0078)	0.0019 (–0.0013, 0.0052)	0.0016 (–0.0021, 0.0053)	0.0032 (–0.0063, 0.0128)
ΣDEHP	0.0002 (–0.0001, 0.0005)	0.0001 (–0.0002, 0.0005)	0.0008 (–0.0008, 0.0025)	0.0004 (–0.0003, 0.0010)	0.0003 (–0.0004, 0.0010)	0.0008 (–0.0023, 0.0039)

Abbreviation: ΣDBP: sum of concentrations of all measured DBP metabolites (MiBP, and MnBP); ΣDEHP, sum of concentrations of all measured DEHP metabolites (MEHP, MEHHP, and MEOHP). * $p < 0.05$.

Table S2. Association of thyroid hormones and urine phthalate metabolites in three groups comparison (without creatinine correction).

	Concentrations (μg/L)	Free T4 (ng/dL)	*p*	Total T4 (μg/dL)	*p*	Free T3 (ng/dL)	*p*	Total T3 (μg/dL)	*p*	TSH (miu/mL)	*p*
**all**											
MEP			0.338		0.860		0.190		0.498		0.767
	<20.07	1.37		8.69		3.98		153.47		2.58	
	20.07–51.41	1.39		8.62		4.15		150.72		2.49	
	>51.41	1.39		8.77		4.10		150.98		2.53	
MiBP			0.113		0.385		0.057		0.188		0.192
	<7.96	1.36		8.58		4.07		156.74		2.77	
	7.96–23.12	1.38		8.64		3.99		148.14		2.29	
	>23.12	1.40		8.86		4.16		150.22		2.53	
MnBP			0.225		0.566		0.172		0.685		0.483
	<8.45	1.36		8.55		4.07		154.65		2.44	
	8.45–15.65	1.38		8.82		4.01		151.03		2.59	
	>15.65	1.40		8.72		4.15		149.57		2.57	
ΣDBP			0.126		0.6238		**0.045** *		0.368		0.512
	<15.86	1.36		8.59		4.07		156.15		2.62	
	15.86–52.78	1.38		8.64		4.00		150.01		2.39	
	>52.78	1.41		8.85		4.16		149.04		2.59	
MEHP			0.085		0.086		0.177		0.641		0.145
	<2.09	1.35		8.42		3.97		150.49		2.75	
	2.09–7.67	1.41		9.00		4.15		155.16		2.28	
	>7.67	1.39		8.67		4.10		149.53		2.58	
MEHHP			0.320		0.234		**0.010** *		0.202		0.635
	<9.71	1.36		8.63		4.02		155.54		2.58	
	9.71–25.58	1.38		8.52		4.01		147.01		2.42	
	>25.58	1.40		8.93		4.19		152.58		2.60	
MEOHP			0.334		0.432		0.104		0.839		0.742
	<6.35	1.36		8.59		4.02		154.20		2.64	
	6.35–16.77	1.38		8.61		4.07		150.19		2.39	
	>16.77	1.40		8.88		4.14		150.82		2.57	
ΣDEHP			0.131		0.352		0.091		0.651		0.584
	<19.09	1.35		8.51		4.04		154.24		2.64	
	19.09–47.87	1.39		8.68		4.03		149.74		2.38	
	>47.87	1.40		8.89		4.16		151.22		2.59	

Table S2. *Cont.*

	Concentrations (μg/L)	Free T4 (ng/dL)	*p*	Total T4 (μg/dL)	*p*	Free T3 (ng/dL)	*p*	Total T3 (μg/dL)	*p*	TSH (miu/mL)	*p*
**boys**											
MEP			0.676		0.277		0.212		0.576		0.209
	<20.07	1.38		8.49		3.98		149.47		2.99	
	20.07–51.41	1.40		8.80		4.18		148.18		2.41	
	>51.41	1.38		9.05		4.12		152.97		2.53	
MiBP			0.754		0.427		0.103		0.686		0.162
	<7.96	1.37		8.60		4.02		153.46		3.04	
	7.96–23.12	1.39		8.77		4.06		148.14		2.32	
	>23.12	1.40		9.07		4.22		149.50		2.50	
MnBP			0.992		0.375		0.054		0.876		0.968
	<8.45	1.39		8.50		4.02		150.73		2.73	
	8.45–15.65	1.38		9.01		3.99		149.15		2.65	
	>15.65	1.39		8.86		4.26		151.49		2.52	
ΣDBP			0.884		0.511		**0.009 ***		0.772		0.218
	<15.86	1.38		8.56		4.00		151.41		3.03	
	15.86–52.78	1.39		8.85		4.02		149.22		2.36	
	>52.78	1.39		9.00		4.26		150.78		2.53	
MEHP			0.180		0.015 *		0.178		0.265		0.233
	<2.09	1.36		8.22		3.97		144.37		3.03	
	2.09–7.67	1.42		9.29		4.14		155.52		2.32	
	>7.67	1.38		8.74		4.14		149.61		2.65	
MEHHP			0.822		0.125		**0.004 ***		0.071		0.867
	<9.71	1.38		8.62		4.04		153.22		2.84	
	9.71–25.58	1.38		8.59		3.95		144.25		2.55	
	>25.58	1.40		9.18		4.28		154.97		2.56	
MEOHP			0.801		0.521		0.218		0.767		0.773
	<6.35	1.38		8.60		4.03		152.38		2.87	
	6.35–16.77	1.39		8.81		4.04		147.67		2.58	
	>16.77	1.39		8.97		4.21		151.67		2.49	
ΣDEHP			0.454		0.251		0.076		0.629		0.593
	<19.09	1.36		8.39		4.03		150.28		2.97	
	19.09–47.87	1.40		8.87		4.01		148.54		2.51	
	>47.87	1.39		9.04		4.25		152.87		2.53	

Table S2. *Cont.*

	Concentrations (μg/L)	Free T4 (ng/dL)	*p*	Total T4 (μg/dL)	*p*	Free T3 (ng/dL)	*p*	Total T3 (μg/dL)	*p*	TSH (miu/mL)	*p*
**girls**											
MEP			0.530		0.307		0.590		0.164		0.610
	<20.07	1.36		8.83		3.98		156.46		2.27	
	20.07–51.41	1.38		8.46		4.12		153.02		2.56	
	>51.41	1.39		8.42		4.09		148.50		2.53	
MiBP			0.107		0.751		0.332		0.186		0.718
	<7.96	1.34		8.56		4.13		160.13		2.50	
	7.96–23.12	1.37		8.51		3.92		148.15		2.27	
	>23.12	1.41		8.68		4.11		150.83		2.56	
MnBP			0.081		0.862		0.948		0.229		0.343
	<8.45	1.34		8.59		4.10		157.48		2.23	
	8.45–15.65	1.38		8.61		4.02		153.09		2.52	
	>15.65	1.41		8.57		4.04		147.54		2.63	
ΣDBP			**0.055** *		0.769		0.835		0.087		0.451
	<15.86	1.34		8.61		4.12		160.19		2.27	
	15.86–52.78	1.36		8.42		3.98		150.90		2.43	
	>52.78	1.42		8.71		4.06		147.45		2.64	
MEHP			0.261		0.998		0.780		0.393		0.375
	<2.09	1.34		8.56		3.98		154.64		2.56	
	2.09–7.67	1.40		8.64		4.15		154.71		2.24	
	>7.67	1.39		8.58		4.07		149.45		2.50	
MEHHP			0.372		0.862		0.258		0.610		0.509
	<9.71	1.35		8.63		4.01		157.00		2.41	
	9.71–25.58	1.39		8.44		4.09		150.60		2.25	
	>25.58	1.40		8.66		4.09		150.11		2.65	
MEOHP			0.339		0.691		0.278		0.698		0.396
	<6.35	1.35		8.58		4.01		155.51		2.47	
	6.35–16.77	1.37		8.39		4.10		153.06		2.18	
	>16.77	1.41		8.77		4.08		149.95		2.67	
ΣDEHP			0.196		0.730		0.615		0.500		0.450
	<19.09	1.34		8.58		4.04		156.57		2.44	
	19.09–47.87	1.37		8.38		4.05		151.63		2.18	
	>47.87	1.41		8.74		4.08		149.71		2.64	

Abbreviation: ΣDBP: sum of concentrations of all measured DBP metabolites (MiBP, and MnBP); ΣDEHP, sum of concentrations of all measured DEHP metabolites (MEHP, MEHHP, and MEOHP). * $p < 0.05$.

Table S3. Association of thyroid hormones and urine phthalate metabolites in three groups comparison (the correction of creatinine).

	**Concentrations (μg/L)**	**Free T4 (ng/dL)**	***p***	**Total T4 (μg/dL)**	***p***	**Free T3 (ng/dL)**	***p***	**Total T3 (μg/dL)**	***p***	**TSH (miu/mL)**	***p***
**all**											
MEP			0.549		0.248		0.440		0.572		0.060
	<23.07	1.38		8.58		4.01		151.29		2.70	
	23.07–50.55	1.38		8.58		4.12		150.50		2.34	
	>50.55	1.39		8.92		4.10		153.30		2.57	
MiBP			0.167		0.424		0.366		0.347		0.744
	<9.49	1.37		8.65		4.07		156.52		2.71	
	9.49–24.19	1.37		8.57		4.05		149.26		2.40	
	>24.19	1.40		8.87		4.11		149.49		2.49	
MnBP			0.079		0.364		**0.047 ***		0.153		0.507
	<9.52	1.36		8.62		4.10		156.50		2.56	
	9.52–26.47	1.38		8.56		3.95		146.84		2.41	
	>26.47	1.41		8.90		4.17		151.68		2.63	
ΣDBP			0.134		0.457		0.487		0.591		0.975
	<23.24	1.36		8.51		4.09		155.54		2.62	
	23.24–52.28	1.39		8.75		4.03		149.98		2.47	
	>52.28	1.40		8.82		4.11		149.74		2.52	
MEHP			0.0880		0.3002		0.3272		0.7072		0.0546
	<2.74	1.36		8.55		4.01		151.07		2.78	
	2.74–6.42	1.40		8.91		4.09		154.37		2.30	
	>6.42	1.38		8.63		4.13		149.82		2.53	
MEHHP			**0.032 ***		**0.043 ***		**0.017 ***		0.857		0.677
	<13.04	1.35		8.42		3.98		152.34		2.60	
	13.04–25.93	1.40		8.94		4.08		151.91		2.45	
	>25.93	1.39		8.74		4.17		150.93		2.55	
MEOHP			0.100		0.076		0.118		0.887		0.602
	<9.15	1.35		8.43		4.02		153.75		2.69	
	9.15–17.95	1.39		8.94		4.06		150.51		2.47	
	>17.95	1.40		8.72		4.15		150.90		2.44	
ΣDEHP			**0.010 ***		**0.010 ***		**0.016 ***		0.779		0.864
	<25.34	1.35		8.36		3.97		151.62		2.65	
	25.34–49.92	1.41		8.98		4.11		152.34		2.50	
	>49.92	1.38		8.75		4.15		151.21		2.45	

Table S3. *Cont.*

	Concentrations (μg/L)	Free T4 (ng/dL)	*p*	Total T4 (μg/dL)	*p*	Free T3 (ng/dL)	*p*	Total T3 (μg/dL)	*p*	TSH (miu/mL)	*p*
**boys**											
MEP			0.781		0.113		0.2876		0.0876		0.1576
	<23.07	1.40		8.53		4.00		146.99		2.92	
	23.07–50.55	1.37		8.67		4.13		146.78		2.39	
	>50.55	1.39		9.21		4.16		157.06		2.52	
MiBP			0.944		0.471		0.192		0.982		0.353
	<9.49	1.38		8.58		3.98		151.79		2.96	
	9.49–24.19	1.40		8.87		4.18		150.36		2.33	
	>24.19	1.38		9.03		4.15		148.80		2.55	
MnBP			0.735		0.270		0.021		0.158		0.762
	<9.52	1.38		8.62		4.03		152.36		2.79	
	9.52–26.47	1.37		8.65		3.96		143.43		2.65	
	>26.47	1.40		9.12		4.27		154.03		2.45	
ΣDBP			0.890		0.229		0.145		0.773		0.716
	<23.24	1.38		8.50		3.99		150.23		2.85	
	23.24–52.28	1.40		9.05		4.13		152.76		2.56	
	>52.28	1.39		8.95		4.20		148.43		2.42	
MEHP			0.058		0.088		0.485		0.712		0.471
	<2.74	1.37		8.49		4.03		147.54		2.93	
	2.74–6.42	1.43		9.24		4.07		153.57		2.41	
	>6.42	1.36		8.70		4.17		150.13		2.57	
MEHHP			0.461		0.109		**0.007 ***		0.189		0.825
	<13.04	1.37		8.39		3.90		145.66		2.85	
	13.04–25.93	1.40		9.02		4.10		152.47		2.56	
	>25.93	1.39		8.98		4.28		152.83		2.49	
MEOHP			0.992		0.456		0.114		0.772		0.212
	<9.15	1.39		8.57		3.98		149.23		2.98	
	9.15–17.95	1.38		8.93		4.07		149.92		2.43	
	>17.95	1.39		8.91		4.24		152.18		2.49	
ΣDEHP			0.193		0.098		**0.021 ***		0.274		0.588
	<25.34	1.37		8.38		3.90		146.20		2.93	
	25.34–49.92	1.42		9.08		4.13		151.74		2.55	
	>49.92	1.37		8.91		4.23		152.87		2.44	

Table S3. *Cont.*

	Concentrations (μg/L)	Free T4 (ng/dL)	*p*	Total T4 (μg/dL)	*p*	Free T3 (ng/dL)	*p*	Total T3 (μg/dL)	*p*	TSH (miu/mL)	*p*
**girls**											
MEP			0.296		0.919		0.585		0.377		0.335
	<23.07	1.35		8.63		4.01		156.51		2.44	
	23.07–50.55	1.38		8.52		4.11		153.19		2.30	
	>50.55	1.39		8.63		4.04		149.65		2.61	
MiBP			**0.024 ***		0.325		0.379		0.098		0.636
	<9.49	1.35		8.73		4.19		162.26		2.41	
	9.49–24.19	1.34		8.28		3.92		148.20		2.47	
	>24.19	1.42		8.75		4.08		150.00		2.45	
MnBP			**0.022 ***		0.888		0.687		0.118		0.100
	<9.52	1.33		8.61		4.18		161.06		2.30	
	9.52–26.47	1.38		8.50		3.95		149.37		2.24	
	>26.47	1.41		8.67		4.07		149.25		2.81	
ΣDBP			**0.037 ***		0.766		0.646		0.097		0.708
	<23.24	1.33		8.52		4.22		162.42		2.32	
	23.24–52.28	1.38		8.51		3.95		147.69		2.38	
	>52.28	1.41		8.72		4.04		150.80		2.60	
MEHP			0.163		0.988		0.521		0.524		0.060
	<2.74	1.35		8.60		3.99		154.18		2.65	
	2.74–6.42	1.38		8.60		4.10		155.11		2.20	
	>6.42	1.40		8.57		4.10		149.50		2.48	
MEHHP			0.056		0.371		0.662		0.399		0.351
	<13.04	1.34		8.45		4.04		158.05		2.39	
	13.04–25.93	1.41		8.83		4.05		151.24		2.32	
	>25.93	1.39		8.53		4.08		149.35		2.60	
MEOHP			**0.019 ***		0.192		0.660		0.355		0.921
	<9.15	1.32		8.29		4.05		157.86		2.43	
	9.15–17.95	1.40		8.95		4.06		151.13		2.51	
	>17.95	1.41		8.56		4.07		149.77		2.40	
ΣDEHP			**0.019 ***		0.185		0.461		0.619		0.837
	<25.34	1.33		8.34		4.02		156.09		2.42	
	25.34–49.92	1.41		8.87		4.08		152.99		2.45	
	>49.92	1.40		8.60		4.08		149.66		2.47	

Abbreviation: ΣDBP: sum of concentrations of all measured DBP metabolites (MiBP, and MnBP); ΣDEHP, sum of concentrations of all measured DEHP metabolites (MEHP, MEHHP, and MEOHP); * $p < 0.05$.

© 2017 by the authors; licensee MDPI, Basel, Switzerland. This article is an open access article distributed under the terms and conditions of the Creative Commons by Attribution (CC-BY) license (http://creativecommons.org/licenses/by/4.0/).
